# Between expectation and reality. A plea for more evidence-based bedside teaching

**DOI:** 10.3205/zma001559

**Published:** 2022-07-15

**Authors:** Thomas Rotthoff

**Affiliations:** 1 University of Augsburg, Faculty of Medicine, Medical Didactics and Educational Research, DEMEDA, Augsburg, Germany

## Editorial

If one asks physicians in Germany about the importance of bedside teaching, the unanimous answer is that it is essential for medical education. Internationally, a similar impression emerges; for example, in a study in the New York metropolitan area, 71% of the physicians surveyed were convinced that bedside teaching should have priority, and 77% were in favor of a stronger emphasis on this teaching format [1]. This emphasized meaning can probably be attributed to physicians’ self-image, which is patient-focused and thus at the focus of teaching and learning. Therefore, it is surprising that the available scientific evidence for this teaching format is very small and prospective randomized studies on bedside teaching have hardly been available in the literature of the last decade [2], [3], [4]. Also, despite its emphasized importance, bedside teaching seems to have come under increasing pressure in its actual implementation in recent years and is at risk of being neglected [5]. This can be derived from recent publications with meaningful titles such as 

*“Anamnesis and physical examination are still indispensable today” *[6]*, *

*“The road back to the bedside” *[7]*,*

and even videos on YouTube in which students ironically cope with their “non-experience” of bedside teaching during their studies [https://youtu.be/heJ5pCMqKoE]. Reasons given in the literature for the decline of bedside teaching include an increased workload for physicians, an increase in imaging procedures and laboratory tests, and technification and digitalization with a greater focus on the computerized image of the patient [8]. Verghese evocatively characterized this as the “iPatient”: 

*“iPatients are handily discussed in the bunker, while the real patients keep the beds warm and ensure that the folders bearing their names stay alive on the computer” *[8]*. *

Changes in the daily routine of physicians result in less contact time between physicians and their patients [9], fewer physical examinations, and ultimately a decrease in clinical practical skills among young physicians [10]. This, in turn, may lead to an increase in errors and misdiagnosis [11]. Today's references to the quote attributed to Sir William Osler (1849-1919), 

*“Medicine is learned by the bedside and not in the classroom” *[12]*, *

seem like an appeal to this background to (re)align wish and reality. 

A further accompanying effect may be fewer medical didactic training opportunities for this teaching format. An own web search on such trainings offered by the German medical faculties, which was carried out at the beginning of 2022, showed that only about 30% of the faculties offer explicit trainings for bedside teaching or teaching in the clinical environment on their websites. Possibly, bedside teaching is regarded as a less stand-alone, challenging, and attainable teaching format compared to other formats such as lecture, seminar, tutorial, or even online courses. Here, physicians are acting in the role of medical experts who “just” perform clinical practice in the presence of students. Didactic weaknesses of teachers in providing bedside teaching, such as

- a focus on facts rather than on the development of problem-solving skills and attitudes,
- too much complexity and too little deconstruction of the patient case,
- more passive observation rather than active participation of learners, and
- insufficient opportunity for reflection, discussion, and feedback,

have been adequately described [13]. 

The special didactical characteristic of beside teaching lies in its cooperative implementation and triangulation of the three groups of participants: doctor, patient, and students. Like no other teaching format, bedside teaching offers the opportunity to link different professional roles of doctors with each other. It is the only format in medical education where, in a real clinical setting, the skills of history-taking and physician-patient communication, physical examination, clinical reasoning, decision-making, empathy, and professionalism can be simultaneously taught and learned as an integrated entity [13], [14]. The medical diagnosis and course of treatment examine the individual case (the individual patient), in which a variety of conditions may be considered [15]. In medicine, interpersonal aspects in particular are conveyed primarily by role models [16] with their attitudes and behaviors [17]. All participants are likewise involved, with the patient being more than just the “demonstrator” of a finding or diagnosis, since patients always comment on and interpret their complaints [15]. Bedside teaching in small groups is a challenging teaching format that, in addition to the aforementioned competencies, also requires a scientifically critical exploration of clinical problems in a special way. In comparison to other teaching and learning formats, it may be postulated that, in addition to strengthening learning and knowledge transfer and improving clinical thinking, the consideration of learning theories [18], metacognitive impulses, scientifically critical examination, activating and systematic methods, and good briefing and debriefing can also strenghten and improve bedside teaching. The teaching format is human resource-intensive and firmly anchored in the German licensing regulations for physicians. 

From a didactic perspective, it therefore seems worthwhile and necessary to bring bedside teaching into stronger focus in educational research and didactic training programs with the creation of new scientific evidence.

In this issue of the GMS Journal for Medical Education, van der Keylen et al. report the rise of digital teaching and learning formats during the COVID-19 pandemic and its high level of acceptance among students [19]. Other articles in this issue however also support the relevance of bedside teaching involving practices from history taking and clinical reasoning to empathizing. In particular, Rahmann et al. highlight the relevance of developing empathy by referring to role modeling and workplace-based learning [20]. Furthermore, Lange et al. point out the students’ high acceptance of an online course to study taking a systematic medical history but however indicating that students consider Blended-Learning to be more efficient [21]. Flugelman et al. suggest a new method of active learning for small groups regarding clinical reasoning, which could be helpful as a preparation or supplement for bedside teaching [22]. At present, the medical workplace is changing and the proportion of women in medicine is increasing, which inevitably coincides with teaching technique and orientation to female behavior for role modelling. Meyer-Frießem et al. and Hege et al. note that leading positions in almost all medical departments as well as medical education are still considerably less often held by female clinicians than by their male counterparts [23], [24]. They suggest mentoring and networking programs and greater consideration being given to woman, leading to them securing positions on the board of directors. Finally, due to a growing focus on interprofessional collaboration in the medical workplace, Ulrich et al. provide suggestions for the designation of teachers in the field of interprofessional education [25]. Against these backgrounds, bedside teaching needs to be refined and further developed, but must by no means fall behind. Ongoing changes in the workplace, such as the increasing proportion of female physicians and more intensive interprofessional collaboration, ultimately also influence bedside teaching with implications for professional identity formation and the development of individual professional roles.

## Competing interests

The author declares that he has no competing interests.
